# Mixed convection in sinusoidal lid driven cavity with non-uniform temperature distribution on the wall utilizing nanofluid

**DOI:** 10.1016/j.heliyon.2021.e06907

**Published:** 2021-05-01

**Authors:** Sattar Aljabair, Ali L. Ekaid, Sahira Hasan Ibrahim, Israa Alesbe

**Affiliations:** Mechanical Engineering Department, University of Technology, Baghdad, Iraq

**Keywords:** Mixed convection, Arc cavity, Lid-driven, Stream vorticity, Nanofluid

## Abstract

Mixed convection heat transfer of Cu-water nanofluid in an arc cavity with non-uniform heating has been numerically studied. The top flat moving wall is isothermally cooled at Tc and moved with a constant velocity. While the heated arc stationary wall of the cavity is maintained at a hot temperature Th. FORTRAN code is used to solve the mass, momentum, and energy equations in dimensionless form with suitable boundary conditions. In this study, the Reynolds number changed from 1 to 2000, and the Rayleigh number changed from 0 to 10^7^. Also, the range of nanoparticles volume fraction extends from ϕ = 0 to 0.07. Stream vorticity method selected for the discretization of flow and energy equations. The present results are compared with the previous results for the validation part, where the results found a good agreement with the others works. The isotherms are regulated near the arc-shape wall causing a steep temperature gradient at these regions and the local and average heat transfer rate increases with increased volume fraction or Reynolds number or Rayleigh number. Finally, Correlation equations of the average Nusselt number from numerical results are presented.

## 1 Introduction

Buoyancy and shear forces are major effective parameters in mixed convection. Where the flow complexity depended on it. Therefore, there are many numerical and experimental studies extended in this field to clear it. Where the important goals of these studies were to the enhancement of heat transfer. In the last years the research path towards to nanofluid media, due to higher thermal conductivity [1]. The research path divides into two parts, heat transfer enhancement [2, 3] or nanofluids thermophysical properties [4, 5, 6, 7, 8].

During nanofluid natural convection case the buoyancy driven force is dominate, where many investigations in this case with different shapes and boundary conditions [9, 10, 11, 12, 13, 14, 15, 16]. There are many of applications of mixed convection in the practical engineering such as cooling of electronic equipment, heat exchangers design, and heat sinks inside channel [17, 18]. To show the effectiveness of nanofluid, lid-driven cavities a good choice to achieve that. As a result, many of researchers investigated the mixed convection in cavities utilize nanofluid or non-Newtonian according to cavity geometry in the recent years [19, 20, 21, 22, 23, 24, 25].

Kahveci et al. (2016), [26] presented the effect of viscosity models of CuO nanofluid in mixed convection lid-driven cavity during constant heat flux. Grashof number, Richardson number, nanofluid volume fraction were the main parameters in this study. Results show that, Pak and Cho viscosity model gives higher value of circulation intensity comparing with Einstein viscosity model, while Einstein viscosity model gives highest value of average Nusselt number. Sheremet et al. (2015), [27] shows a numerical steady nanofluid mixed convection in lid-driven square cavity by Buongiorno's mathematical model. Where both top and bottom walls were moving with constant temperatures wile vertical walls are insulated. The results included of streamlines, isotherms and iso-concentrations.

Ismael (2017), [28] studied lid driven flow with arc vertical moving wall in both aiding and opposing directions. When Rayleigh numbers were low the heat transfer enhancement irrespective to rotation direction while for high Rayleigh numbers it enhances in the opposing clockwise rotation.

Kapil et al. (2019), [29] used numerical modeling of Al_2_O_3_ nanofluid to study concentration effect. Where Grashof number was fixed at 10^4^ while Reynolds number changed from 1 to 100. Results show that, Nusselt number increase with increase Reynolds number and concentration value. Heated triangular block in lid cavity was presented by [30, 31], this case study bases on block location, block size, Richardson number and Reynolds number. Results found that, increase Ri number leads to increase Nu. Entropy generation of Al_2_O_3_ nanofluid mixed convection in lid driven cavity was presented numerically by Gibanov et al. (2018), [32]. The modeling included the single-phase assumption, Brownian diffusion effect, Richardson number, thermal conductivity ratio, wall thickness and volume fraction. Where the heat transfer enhancement increases with increased volume fraction with reduction in Bejan number. Selimefendigil et al. (2019), [33] presented inclined magnetic field effect on CuO nanofluid in square lid cavity with fin attached the upper wall. Where average heat transfer coefficient increases about 28.96% when volume fraction equal 5% compared to base flued case.

Rahman et al. (2018), [34] used finite element method to modeling the mixed convection lid driven cavity. Where Cu nanoparticles was selected in this study and water as base fluid. Horizontal walls kept insulated while vertical walls kept at isothermal temperature difference. The results cover the range of Richardson number from 0.1 to 10, moreover to Nusselt number and Sherwood number. Wavy wall geometry and entropy generation effects of lid cavity filled with Cu nanofluid presented by Cho et al. (2018), [35] Where high temperature fixed on flat left wall (moves vertically) while low temperature value fixed on wavy right wall (stationary), and other walls were insulated. The results show that, increase Richardson number, volume fraction and Reynolds number leads to increase average Nusselt number and total entropy generation. The Bejan number increases when Re increases, and it reduce when the irreversibility distribution ratio increases.

Abu-Nada et al. (2014), [36] study laminar mixed convection of lid driven wavy cavity filled with CuO nanofluid. Vertical walls were kept insulated while horizontal walls were kept at isothermal temperatures. Top wall was heated and moving with constant velocity while bottom wall was fixed and at cold temperature. Numerical solution with stream function–vorticity formulation was applied to solve the problem. Bottom wall geometry ratio, Ri, and volume fraction were the major parameters in this study. It is found that the presence of nanoparticles causes significant heat transfer for all values of the parameters.

Double side lid driven enclosure with nanofluid investigated numerically using finite element method by Sheikholeslami et al. (2016), [37]. Hot wall selected as a sinusoidal shape function while other walls were flat. The study included the effect of flow and magneto-hydrodynamics with different parameters such as Hartmann, magnetic and Reynolds numbers. The heat transfer enhancement depended directly with Reynolds and Hartmann numbers. A similar analysis of lid-driven cavity of mixed convection with wavy wall and different boundary conditions were presented by [38, 39, 40, 41].

Khajeh et al. (2018), [42] presented laminar Al_2_O_3_ nanofluid flow over cylinder with different cross section, the results focused on convective heat transfer coefficient and the entropy generation. Entropy generated and nanoparticles volume fraction enhanced heat transfer according to the results. Where the highest entropy generation accrue in the vertical elliptical cross section case, with force flow is perpendicular to the buoyant flow force in all mixed convection modes. Sivasankaran et al. [43, 44] presented numerical study of sinusoidal temperature boundary wall on double-diffusive mixed convective in square cavity. Vertical walls kept in Sinusoidal variation of temperature while horizontal walls are adiabatic. Finite-volume method used to solve the problem in transient case, phase deviation, buoyancy ratio, Richardson number and amplitude ratio are computed. Buongiorno's nanofluid model used in lid-driven cavity by Elshehabey et al. (2015), [45]. The study included the effects of Brownian motion and thermophoresis with wide range of Richardson number, buoyancy ratio thermophoresis, Prandtl number, Brownian motion parameter, Amplitude phase angle, inclined magnetic field angle, and Hartmann number. Results demonstrate that, the flow movement was loss when magnetic field was existed.

Finally, many of researcher reported nanofluids mixed convection with lid driven cavity case for different boundary conditions such as [46, 47, 48, 49, 50], where has not enough totally. Therefore, the aim of the current study is to extend and investigate of the nanofluid mixed convection problem in a sinusoidal lid-driven arc cavity with sinusoidal variation of temperature along arc bottom wall.

## 2 Mathematical model

### 2.1 Governing equations and formulation

**Figure 1:**
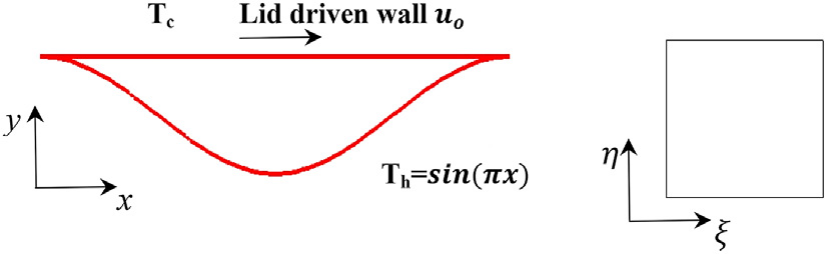
A schematic view and case study description of the physical domain (left) and computational domain (right).

Consider a cold top moving wall with a constant velocity and hot bottom arc wall of the cavity where the heating profile was non uniform sinusoidal equation, as shown in Figure 1 The cavity is filled with Cu–water nanofluid, it was assumed to be Newtonian, incompressible, and laminar and to have constant thermos-physical properties as shown in Table 1, with the exception of density which varies according to the Boussinesq approximation. The base fluid and nanoparticles were assumed to be in thermal equilibrium. The mathematical model of present work was employed to analyze the flow pattern and heat transfer parameters in sinusoidal arc cavity. Under these assumptions, dimensionless governing equations in vorticity-stream function formulation take the following form:

**Table 1:** Thermal physical properties of Cu nanoparticle and water [38].

Material	$C_{p}\left(J/kg.K\right)$	$\rho \left(kg/m^{3}\right)$	k(W/m.K)	β(1/K)
Water	4179	997.1	0.613	21∗10^-5^
Cu	385	8933	400	1.67∗10^-5^

The thermo-physical properties of the nano-fluid are given as shown in Table 1 [38]. Also, for the nanofluid properties are estimated as follows in Table 2 [16].

**Table 2:** Formula model of thermal physical properties of nanofluid [16].

Nanofluid properties	Formula model
Density $\left(kg/m^{3}\right)$	$\rho_{nf}=\left(1-\varphi \right){\left(\rho \beta \right)}_{f+}\varphi \rho_{p}$ (1)
Heat capacity (J/kg.K)	${\left(\rho c_{p}\right)}_{nf}=\left(1-\varphi \right){\left(\rho c_{p}\right)}_{f+}\varphi {\left(\rho c_{p}\right)}_{p}$ (2)
Thermal expansion coefficient (1/K)	${\left(\rho \beta \right)}_{nf}=\left(1-\varphi \right){\left(\rho \beta \right)}_{f+}\varphi {\left(\rho \beta \right)}_{f}$ (3)
Thermal conductivity (W/m.K)	$k_{nf}=k_{f}\frac{\left(k_{s}+2k_{f}\right)-2\phi \left(k_{f}-k_{s}\right)}{\left(k_{s}+2k_{f}\right)+\phi \left(k_{f}-k_{s}\right)}$ (4)
Thermal diffusivity $\left(m^{2}/s\right)$	$\alpha_{nf}=k_{nf}/{\left(\rho c_{p}\right)}_{nf}$ (5)

The governing equations are given in Eqs. (1), (2), (3) using dimensionless vorticity (Ω), stream function (Ψ), and dimensionless temperature (Θ), with dimensionless numbers as shown below (see Table 3):

$$\begin{array}{l} \text{Dimensionless}\\ \text{parameters} \end{array}\;|\;\begin{array}{l} X=\frac{x}{L},Y=\frac{y}{L},U=\frac{u.L}{\alpha_{f}},V=\frac{v.L}{\alpha_{f}},\Omega =\frac{\omega .L^{2}}{\alpha_{f}},\Psi \frac{\psi }{\alpha_{f}}\\ \Theta =\frac{T-T_{c}}{T_{h}-T_{c}},P=\frac{p}{\rho_{nf}.u^{2}},\\ Gr=g\beta \left(T_{h}-T_{c}\right)L^{3}/\vartheta^{2},Re=u_{o}L/\vartheta ,Pr=\vartheta /\alpha \end{array}$$

**Table 3:** Governing equations in different forms.

	In N.S form	In Stream-Vorticity form
Continuity equation	$U_{X}+V_{Y}=0$ (6)	
Momentum in x-direction	$U.U_{X}+V.U_{Y}=-P_{X}+\frac{1}{Re}\left(\frac{\rho_{f}}{\rho_{nf}}\frac{\mu_{nf}}{\mu_{f}}\right)\cdot \nabla^{2}U$ (7)	$\Psi_{XX}+\Psi_{YY}=-\Omega$ (10)
Momentum in y-direction	$\rho_{nf}\left[U.V_{X}+V.V_{Y}\right]=-P_{Y}+\frac{1}{Re}\left(\frac{\rho_{f}}{\rho_{nf}}\frac{\mu_{nf}}{\mu_{f}}\right)\cdot \nabla^{2}V+\frac{Ra}{Pr.Re^{2}}\frac{{\left(\rho \beta \right)}_{nf}}{\rho_{nf}\beta_{f}}\Theta$ (8)	$\Psi_{Y}.\Omega_{X}-\Psi_{X}.\Omega_{Y}=\frac{1}{Re}\left(\frac{\rho_{f}}{\rho_{nf}}\frac{\mu_{nf}}{\mu_{f}}\right)\left(\Omega_{XX}+\Omega_{YY}\right)+\frac{Ra}{Pr.Re^{2}}\frac{{\left(\rho \beta \right)}_{nf}}{\rho_{nf}\beta_{f}}\Theta_{X}$ (11)
Energy equation	$U.\Theta_{X}+V.\Theta_{Y}=\frac{1}{RePr}\frac{\alpha_{nf}}{\alpha_{f}}.\nabla^{2}\Theta$ (9)	$\Psi_{Y}.\Theta_{X}-\Psi_{X}.\Theta_{Y}=\frac{1}{RePr}\frac{\alpha_{nf}}{\alpha_{f}}\left(\Theta_{XX}+\Theta_{YY}\right)$ (12)

Then, based on the body-fitted curvilinear coordinate (ξ,η), the set of Eqs. (10), (11), (12) are written in the transformation form as given below [51]:

(13) $$Α.\Psi_{\xi \xi }+2Β.\;\Psi_{\xi \eta }+C\;\Psi_{\eta \eta }.+D\;\Psi_{\eta }.+E\;\Psi_{\xi }=-J\Omega$$

(14) $$\Psi_{\eta }\;\Omega_{\xi }-\Psi_{\xi }\;\Omega_{\eta }=\frac{1}{Re}\left(\frac{\rho_{f}}{\rho_{nf}}\frac{\mu_{nf}}{\mu_{f}}\right)\left(Α\;\Omega_{\xi \xi }+2Β\Omega_{\xi \eta }+C\Omega_{\eta \eta }+D\Omega_{\eta }+E\Omega_{\xi }\right)+\frac{Ra}{Pr.Re^{2}}\frac{{\left(\rho \beta \right)}_{nf}}{\rho_{nf}\;\beta_{f}}\left[Y_{\eta }\;\Theta_{\xi }-Y_{\xi }\;\Theta_{\eta }\right]$$

(15) $$\Psi_{\eta }\;\Theta_{\xi }-\Psi_{\xi }\;\Theta_{\eta }=\frac{1}{RePr}\frac{\alpha_{nf}}{\alpha_{f}}.\left(Α\;\Theta_{\xi \xi }+2Β\;\Theta_{\xi \eta }+C\;\Theta_{\eta \eta }+D\;\Theta_{\eta }+E\;\Theta_{\xi }\right)$$

Where

$Α=\frac{\Gamma }{J}$, $\;B=\frac{\sigma }{J}$, $C=\frac{\gamma }{J}$, $D=B_{\xi }+\;C_{\eta }$, $\;E=A_{\xi }\;+\;B_{\eta }\;,$ $\Gamma =X_{\eta }^{2}+Y_{\eta }^{2},$
$\;\gamma =X_{\xi }^{2}+Y_{\xi }^{2}$, $\;J=X_{\xi }\;.\;Y_{\eta }-Y_{\xi }\;.\;X_{\eta }$, $\sigma =X_{\xi }\;.\;X_{\eta }+Y_{\xi }\;.\;Y_{\eta }$

### 2.2 Boundary conditions

The boundary conditions on the cold moving wall and the hot sinusoidal arc wall are given in Eqs. (16) and (17), respectively:

(16) $$U=1,\;V=0,\;\text{Ψ}=0,\;\Theta =0,\;\text{Ω}=-\left(1/J\right)\left(U_{\eta }.X_{\xi }\right)\;$$

(17) $$U=0,\;V=0,\;\text{Ψ}=0,\;\Theta =\sin\left(\pi X\right),\;\text{Ω}=-\left(1/J\right)\left(V_{\eta }.Y_{\xi }+U_{\eta }.X_{\xi }\right)\;$$

The local and average Nusselt numbers for the walls are given in the following equations:

(18) $$Nu_{l}=\left(k_{nf}/k_{f}\right)\Theta_{n}$$

(19) $$Nu_{ave}=\frac{1}{S}\overset{S}{\underset{0}{\int }}Nu_{L}ds$$

Where, n is the normal direction to the wall, and s is the wall length.

Finally, iterative method used to couple the stream function and vorticity fields to converged the solution.

### 2.3 Grid generation and grid independence

**Figure 2:**
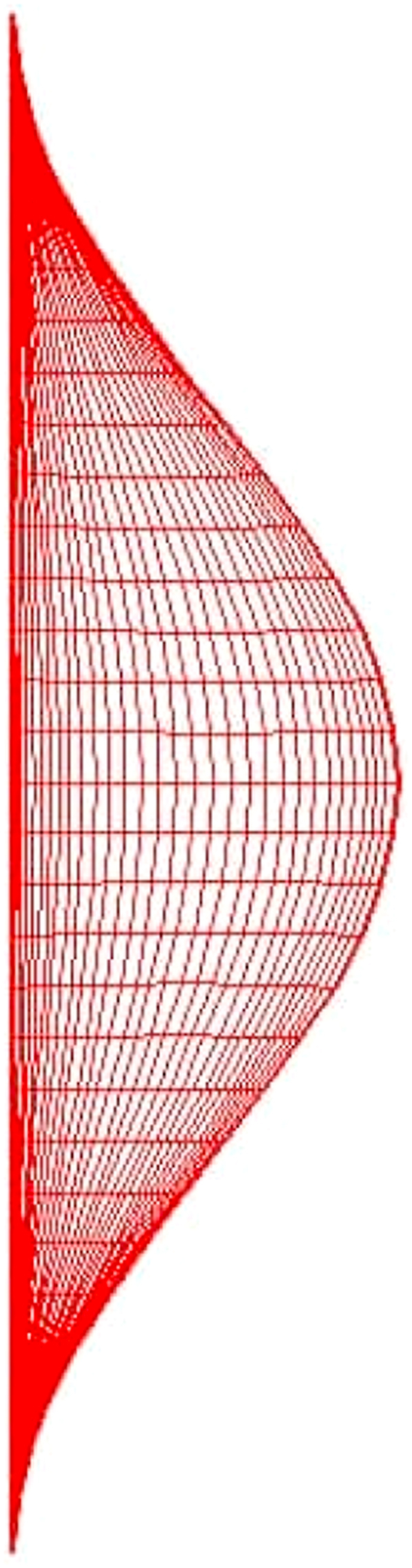
Grid generation of the physical domain.

**Figure 3:**
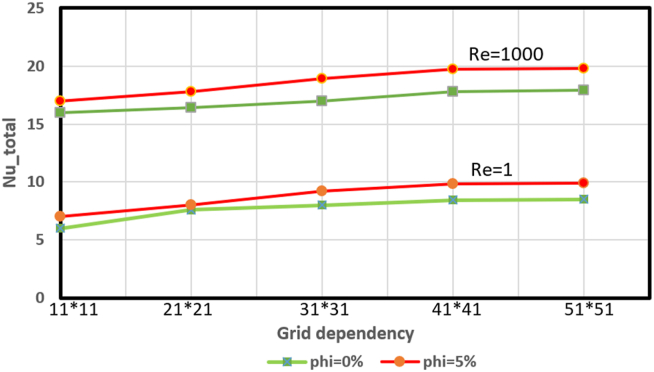
Grid independency tests.

The grid generation technique is based on the curvilinear coordinate system (ξ,η). Where the coordinate transformation functions (i.e., ξ=ξ(X,Y) and η=η(X,Y)) were proposed by Thompson et al. [51] and adopted by Chen, et al. [52]. The transformation functions ξ=ξ(X,Y) and η=η(X,Y) are obtained separately by solving the following elliptic Poisson equations, as given below:

(20) $$\nabla^{2}\xi =P\left(X,Y\right),\;\nabla^{2}\eta =Q\left(X,Y\right)$$

where P and Q are two arbitrary control functions specified to adjust the local density of the grids. Figures 2 and 3 show grid generation and grid dependency in this study.

The governing equations associated with boundary conditions were solved numerically by the finite difference schemes. Central difference and forward and backward upwind difference approximations were utilized for partial differential derivatives and convective terms; respectively. While the line successive over-relaxation L.S.O.R method was used for velocity and temperature fields. This method translated the elliptic partial differential equations into algebraic equations by discretization form with linear Gauss reduction scheme. The stream function calculations were performed by using the successive over-relaxation (SOR) method with tolerance 10^−6^.

### 2.4 Code validation test

**Figure 4:**
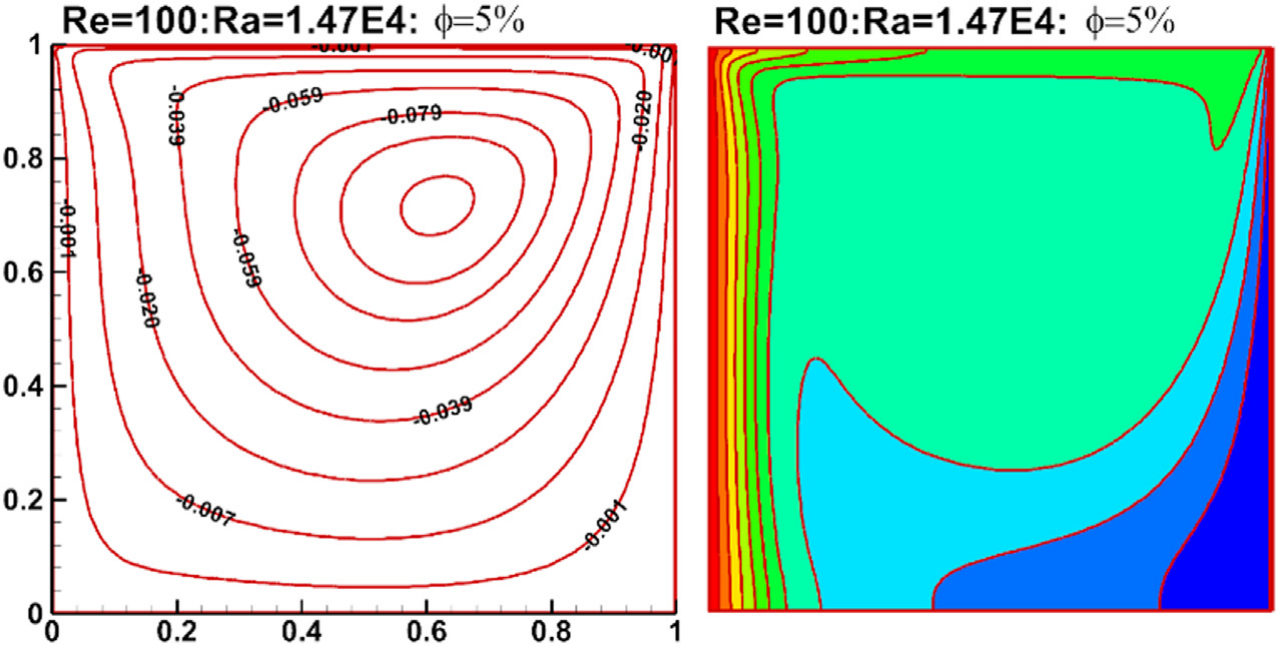
Validation case with Farhad et al. [53] and present results, for stream function (left) and temperature counter (right).

In order to validate the numerical results, a validation test was applied. The present results are compared with the results of Farhad et al. [53]. Validation test presented for mixed convection of Cu–water nanofluid in a lid cavity. The results show a good agreement as shown in Figure 4. The comparison results of $Nu_{ave}$ with Farhad et al. [53] are presented in Table 4.

**Table 4:** Comparison of results obtained in this study by Farhad et al. [53].

Re = 1									
φ	Ra = 1.47∗10^6^			Ra = 1.47∗10^5^			Ra = 1.47∗10^4^		
	[53]	Present results	Error %	[53]	Preset results	Error %	[53]	Present results	Error %
0.0	10.29	10.33	0.38	5.38	5.41	0.52	2.98	3.00	0.61
0.01	11.41	11.62	1.80	5.74	5.80	1.06	3.23	3.25	0.64
0.02	12.46	12.75	2.29	6.17	6.23	1.01	3.33	3.36	0.86
0.03	13.58	13.87	2.10	6.60	6.68	1.25	3.54	3.58	1.07
0.04	14.66	15.01	2.32	7.03	7.16	1.88	3.72	3.77	1.42
0.05	15.67	16.12	2.77	7.45	7.60	1.91	3.93	3.99	1.57
**Re = 10**									
0.0	10.55	10.61	0.54	6.05	6.10	0.81	4.10	4.11	0.28
0.01	11.70	11.81	0.94	6.38	6.45	1.11	4.30	4.34	0.93
0.02	12.75	12.88	1.00	6.89	6.98	1.23	4.47	4.51	0.89
0.03	13.87	14.01	1.03	7.32	7.41	1.27	4.67	4.72	1.03
0.04	14.98	15.33	2.28	7.64	7.75	1.37	4.87	4.93	1.15
0.05	16.03	16.40	2.25	8.16	8.28	1.44	5.07	5.15	1.47
**Re = 100**									
0.0	11.30	11.38	0.68	7.27	7.33	0.78	6.39	6.43	0.56
0.01	12.55	12.66	0.86	7.76	7.83	0.85	6.82	6.89	0.95
0.02	13.77	13.93	1.15	8.28	8.37	1.02	7.16	7.30	1.87
0.03	14.96	15.15	1.27	8.78	8.91	1.51	7.56	7.66	1.26
0.04	16.15	16.60	2.74	9.30	9.48	1.93	7.90	8.02	1.46
0.05	17.33	17.80	2.62	9.76	9.96	2.03	8.21	8.34	1.53

## 3 Results and discussion

### 3.1 Streamlines and isotherms

**Figure 5:**
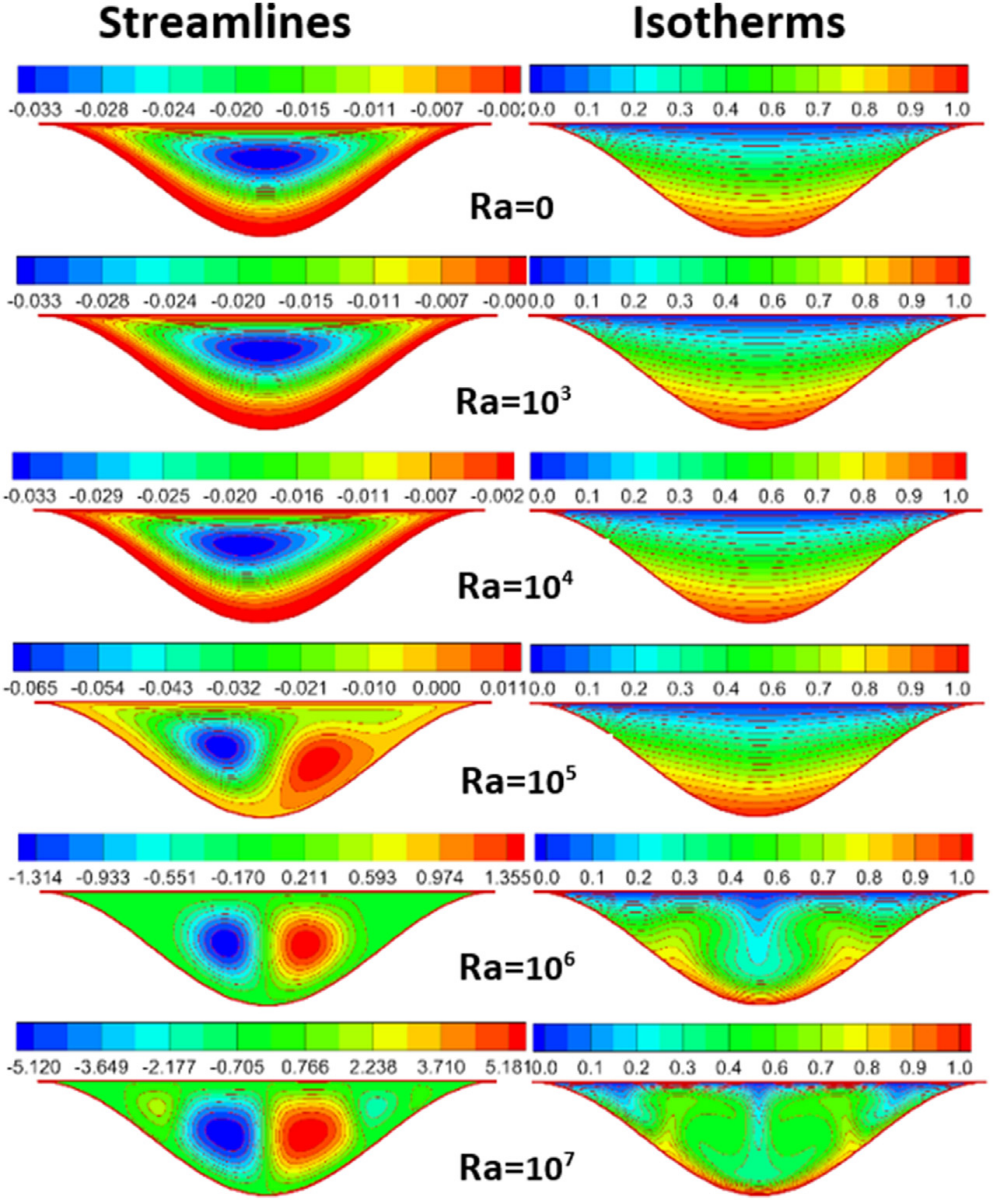
Effect of Rayleigh number on stream function and temperature contour at Re = 1, φ = 3%.

**Figure 6:**
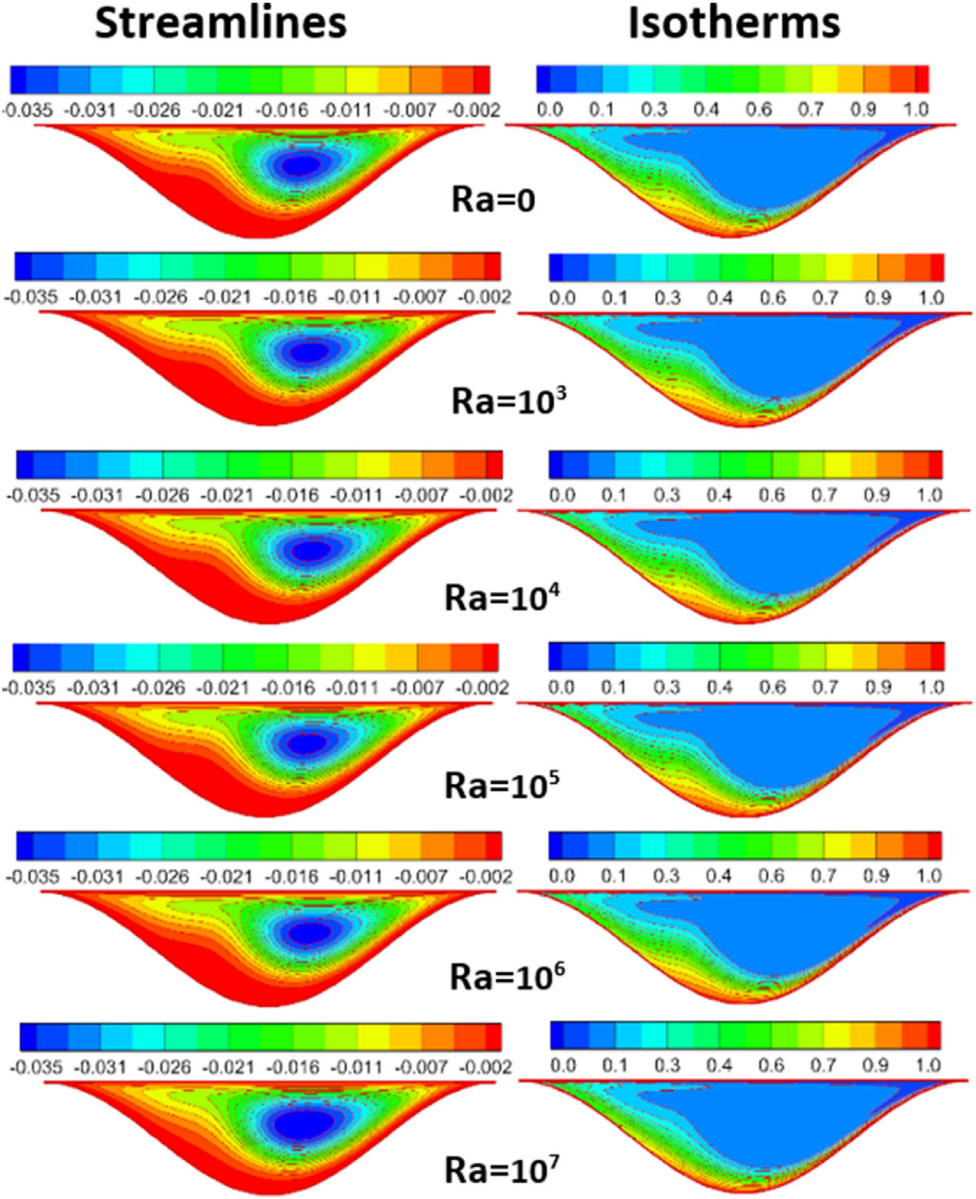
Effect of Rayleigh number on stream function and temperature contour at Re = 2000, φ = 3%.

**Figure 7:**
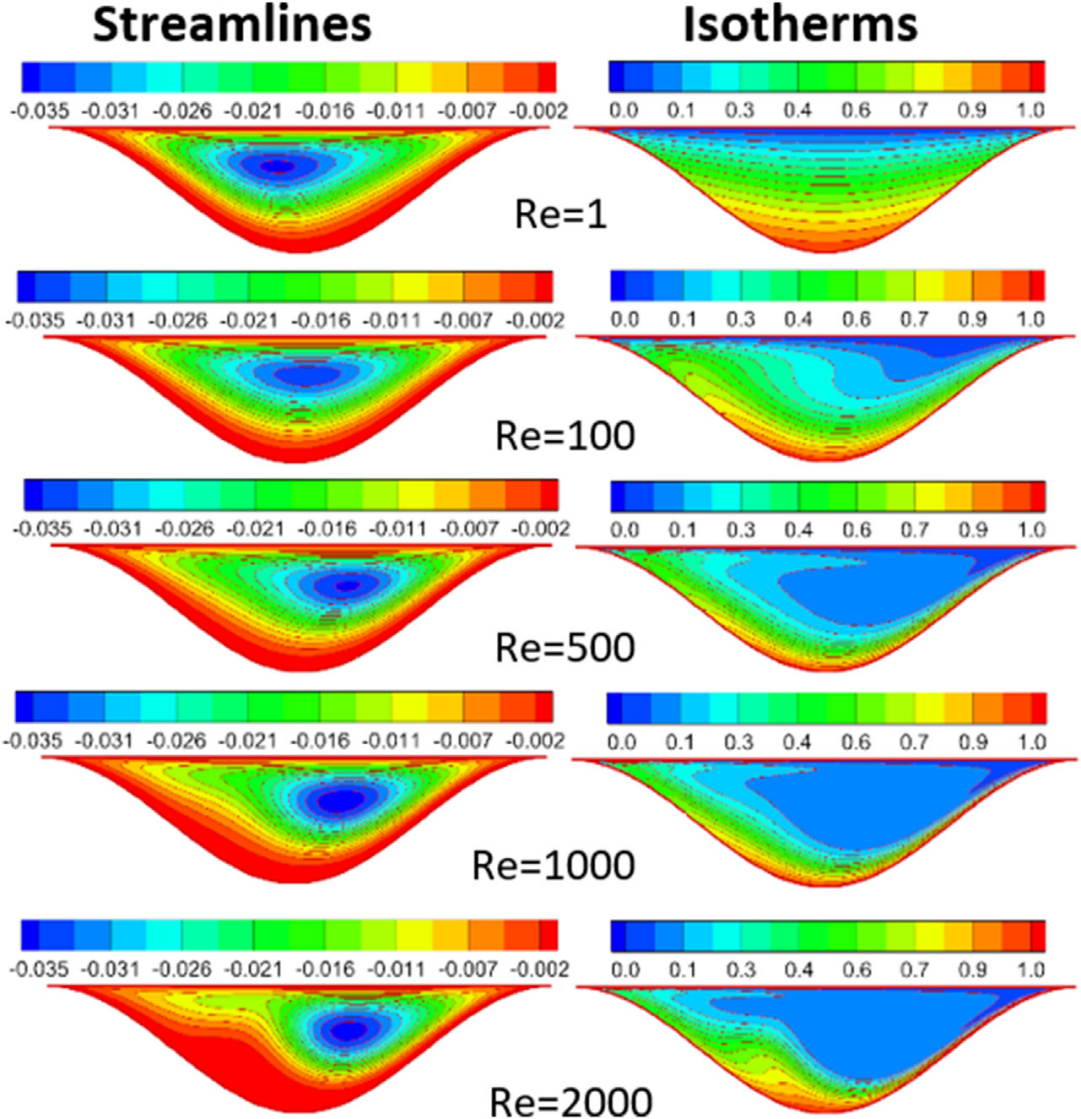
Reynolds number effect on distribution of Stream function and Temperature contour for φ = 1%.

**Figure 8:**
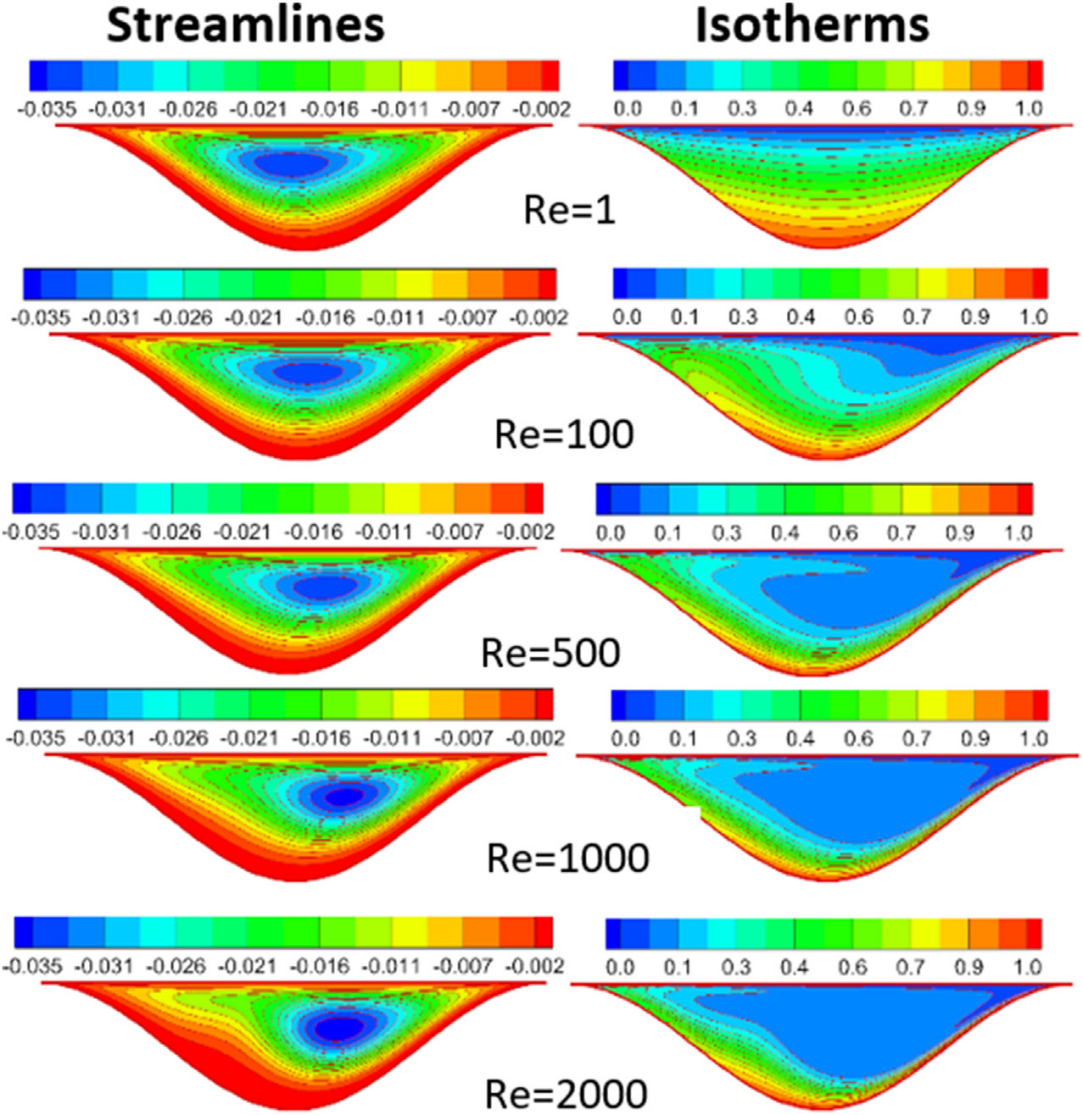
Reynolds number effect on distribution of Stream function and Temperature contour for φ = 5%, Ra = 10^4^.

**Figure 9:**
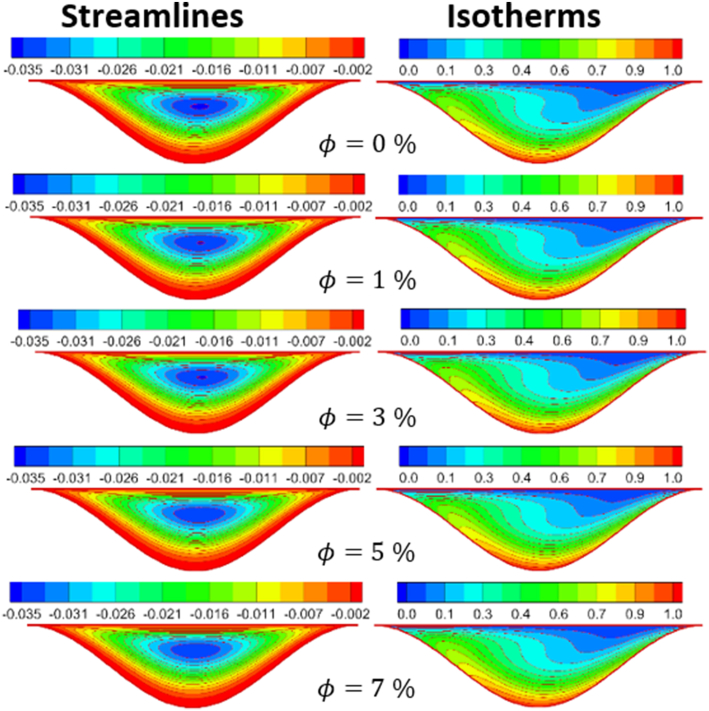
Volume fraction effect on distribution of Stream function and Temperature contour for Re = 100 and Ra = 10^5^.

**Figure 10:**
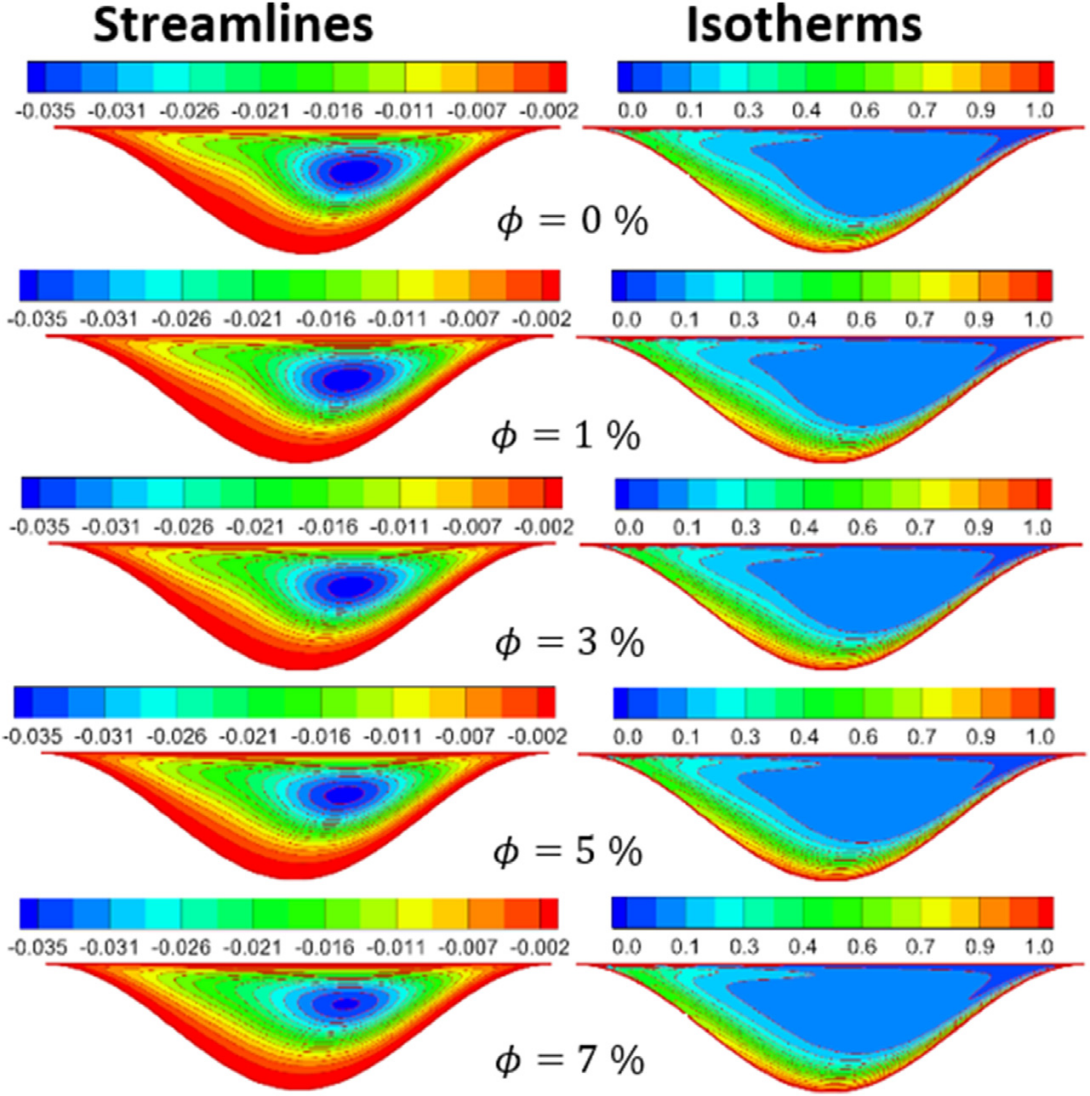
Volume fraction effect on distribution of Stream function and Temperature contour for Re = 1000 and Ra = 10^5^.

The effect of Rayleigh number, Reynolds number and nanoparticles volume fraction on streamlines and isotherms inside lid-driven arc cavity filled with Cu nanofluid are presented in Figures 5, 6, 7, 8, 9, and 10. There is a central flatus vortex located in the central of the cavity for Ra < 10^4^ as shown in Figure 5. While it divided into two vortices and becomes as a circular shape nearly for Ra > 10^4^. The lowest stream strength accrued close to the left top lid wall while the highest stream strength accrued close to the right top lid wall. Isotherms couture is regulated in case one (Ra < 10^4^) and it became mor random when Ra increase and causing a steep temperature gradient near to arc wall, also, the cavity cooling effect becomes clearer. Increasing Re as shown in Figure 6, leads to different thermal flow behavior. In this case the vortex becomes more flatus in the cavity and diffused in the opposite direction of lid moving. In the other hand, the isotherms counter is more deform towards the opposite lid direction and high cooling region in the cavity due to the high inertia force comparing with bouncy force. For low nanoparticles volume fraction φ=1%, the phenomena are the same according to streamlines and isotherms as shown in Figure 7, but when nanoparticles volume fraction increase there are a little change in the thermal performance in the cavity as shown in Figure 8. The flow motion will be more identical in the cavity when there is an equilibrium between the inertia and bouncy force as shown in Figure 9. Also, increasing the volume fraction value leads to beast thermal performance in the cavity. But when the inertia force increasing comparing with bouncy force the flow motion changed and the vortex moved gradually towards to left with lid moving wall direction as shown in Figure 10.

### 3.2 Local and average Nusselt number

**Figure 11:**
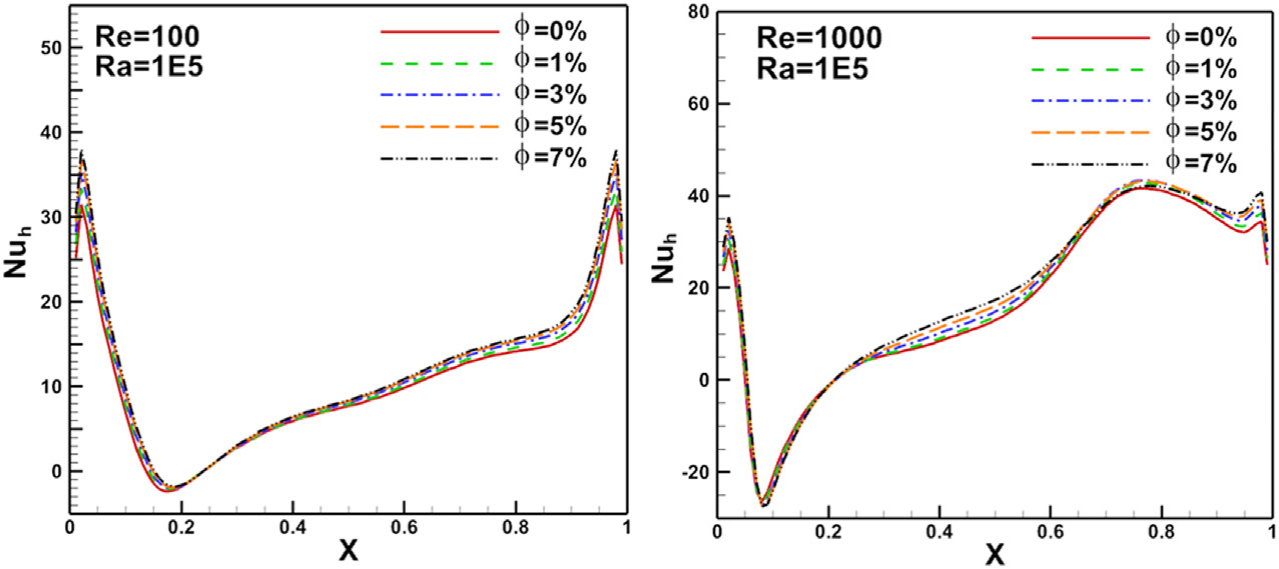
Volume fraction effect on N_u_ distribution on heated arc stationary wall.

**Figure 12:**
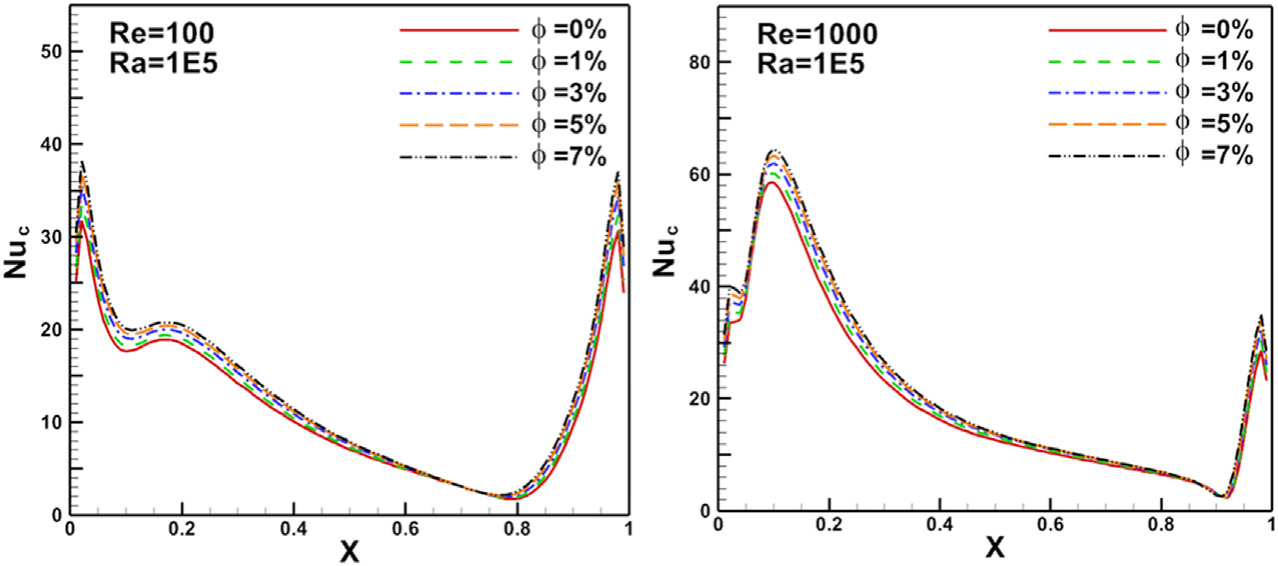
Volume fraction effect on N_ul_ distribution on flat cold moving wall.

**Figure 13:**
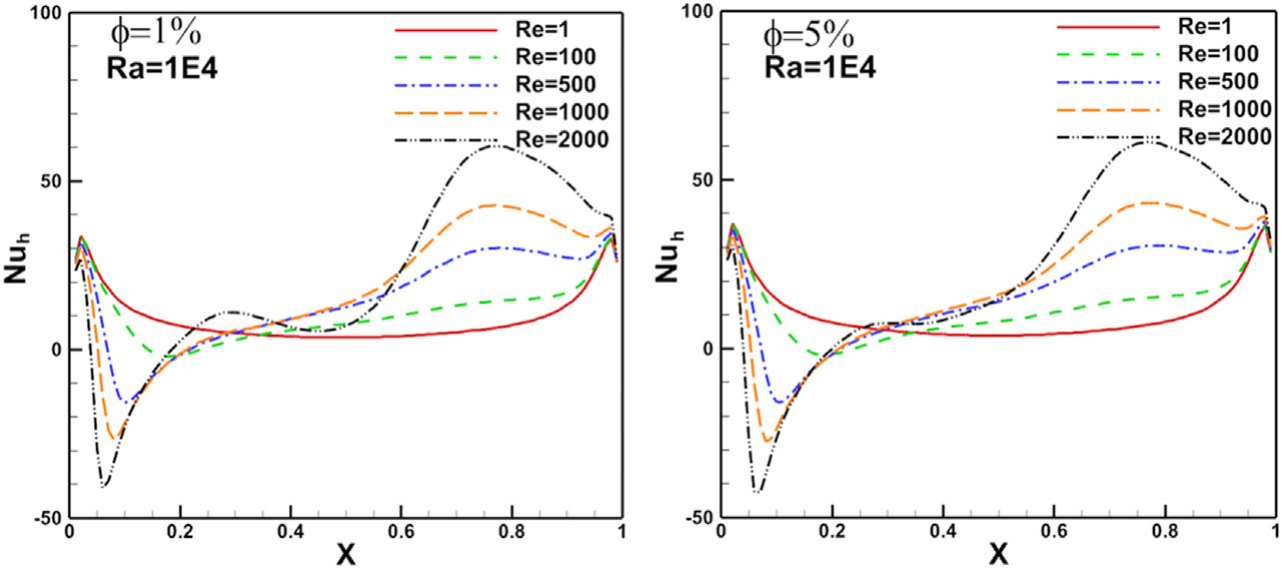
Reynolds number effect on N_ul_ distribution on heated arc stationary wall.

**Figure 14:**
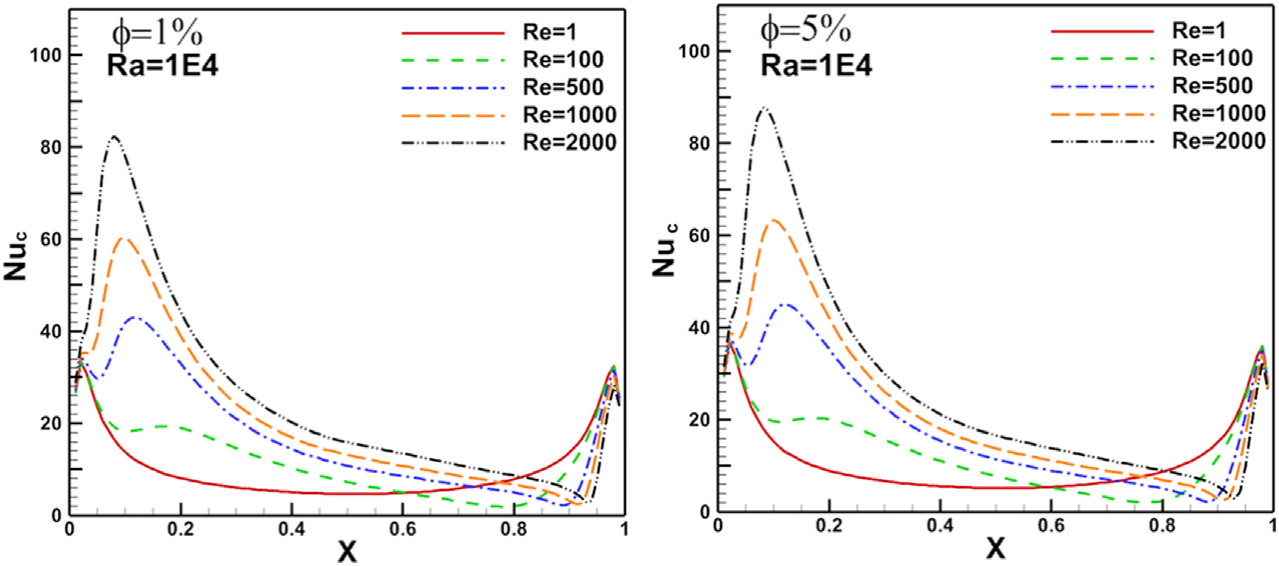
Reynolds number effect on N_ul_ distribution on for flat cold moving wall.

**Figure 15:**
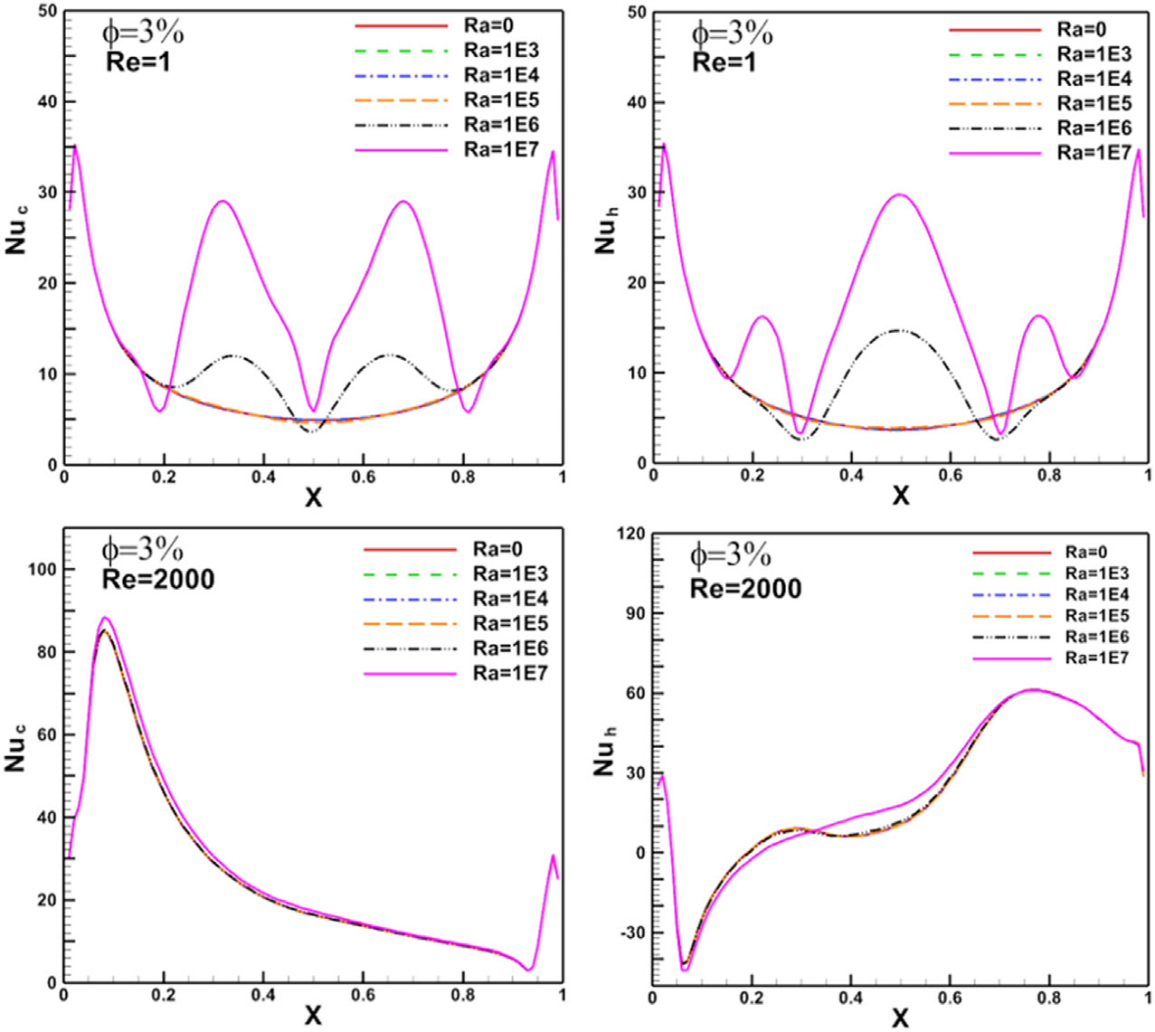
Rayleigh number effect of N_ul_ distribution on flat cold moving wall (left) and heated arc stationary wall (right).

Local Nusselt number distribution on heated arc stationary wall and flat cold moving wall for different nanoparticles volume fraction, Re, and Ra are presented in Figures 11, 12, 13, 14, and 15. Figure 11 illustrates $Nu_{l}$ distribution over heated arc stationary wall for different volume fraction values where Ra fixed at 10^5^ and

**Figure 16:**
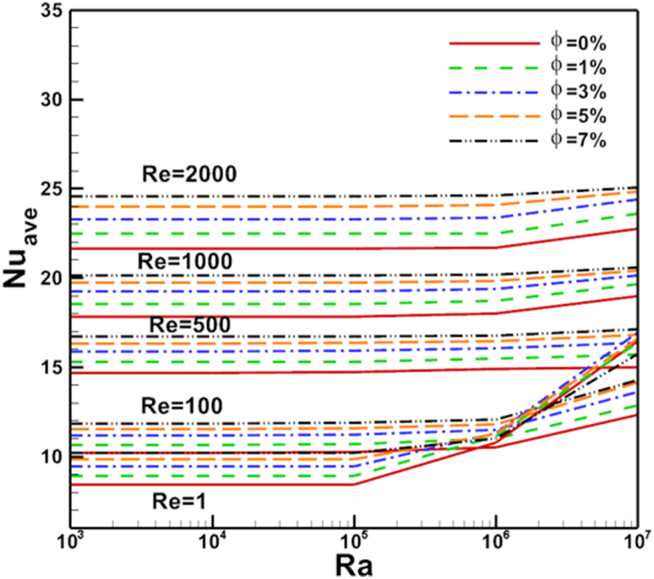
Volume fraction effect of N_uave_ on heated arc stationary wall.

Re changed from 100 to 1000. The results show a good enhancement in heat transfer prosses when nanoparticles increased in the base fluid. The $Nu_{l}$ profile was irregular about the arc wall due to the vortex generation in the domain where it depends on vortex location. The same parameters are presented for flat cold moving wall as shown in Figure 12. Where the effect of increasing Re value is very clear on Nusselt number. Figure 13 presents $Nu_{l}$ distribution over heated arc stationary wall for different Re and volume fraction value. The results show that, increasing Re or volume fraction leads to increasing of $Nu_{l}$ at fixed Ra = 10^4^ according to the increased inertia force in the domain. This behavior repeated with the same heat transfer enhancement in Figure 14 for $Nu_{l}$ over flat cold moving wall. Figure 15 shows Ra effect of $Nu_{l}$ distribution on flat cold moving wall and heated arc stationary wall. Where the profile was symmetry in the domain nearly at low Re, but it will be more random if Re increase due to increased flow strength, moreover to increased $Nu_{l}$ with increased Ra for all cases. Finally, average Nusselt number distribution with different Ra, Re, and φ are shown in Figure 16. In general, for $Ra\leq 10^{5}$ there is small response to change the $\bar{Nu}$ value for all values of Re. In this case, there is an equilibrium between inertia force and bouncy force but if Ra increase more, the $\bar{Nu}$ jamb suddenly to high value.

### 3.3 Correlation equation

The average Nusselt number is correlated with the $Ra/Re^{2}$ and particle volume fraction. Two equations are given for *φ* = 0%, and *φ* = 1%, to 7%, where Ra changed from 0 to 10^7^, Re changed from 1 to 2000 respectively. Least-squares curve fittings of the obtained numerical data have the following correlation form:

$$Nu_{ave}=a*{\left(\mathrm{Ra}/Re^{2}\right)}^{b}*\varphi^{c}$$

**Figure 17:**
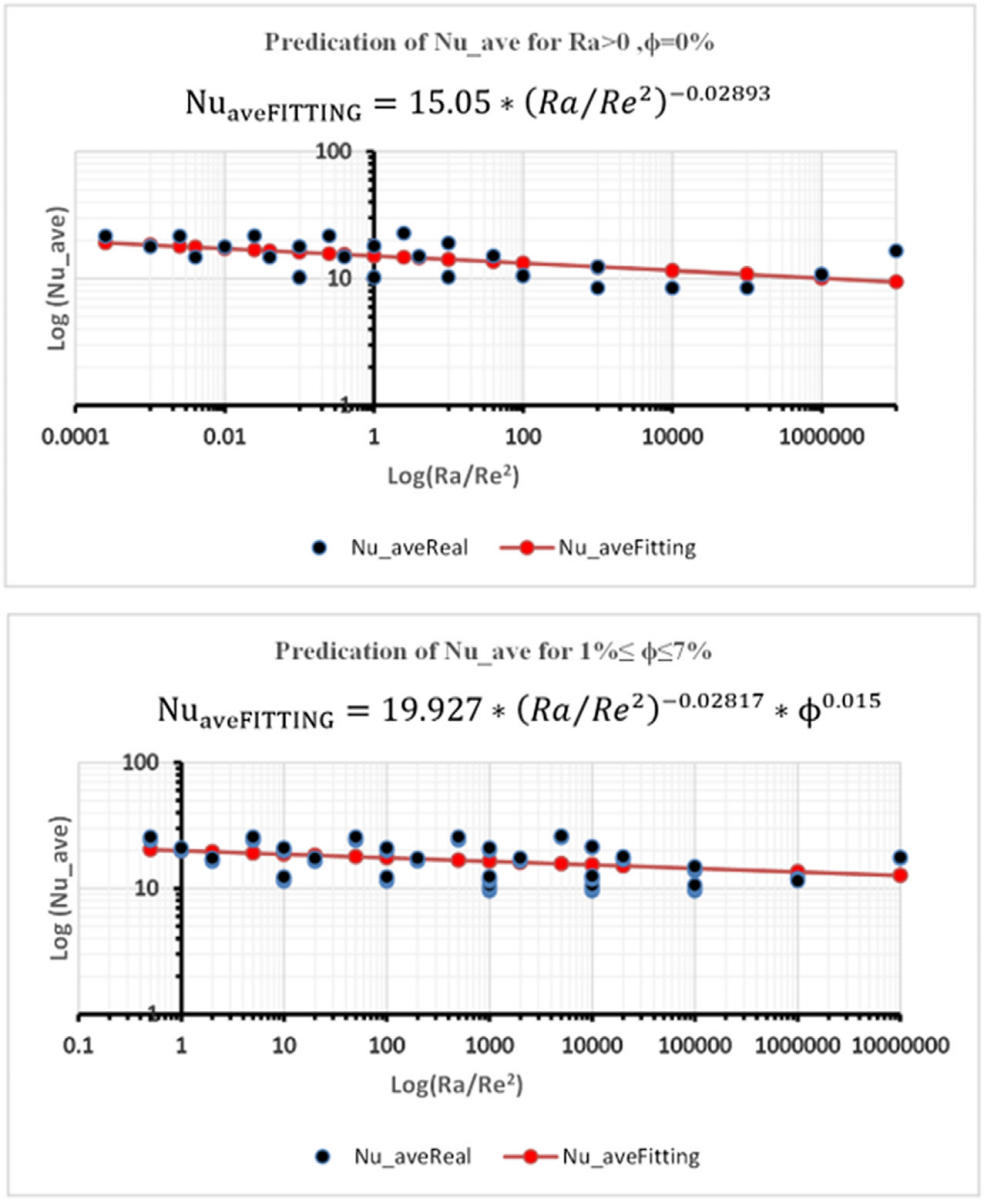
Correlation equations of N_uave_ from numerical results.

Correlation equations of average Nusselt number from numerical results are presented in Figure 17.

## 4 Conclusion

Mixed convection in sinusoidal lid driven cavity with non-uniform temperature distribution on the wall utilizing Cu nanofluid are presented. At lower Gr number the inertia force is dominated and a strong clockwise vortex is driven by the moving lid nearly in the middle of the cavity. Where it moves to the right (with lid direction) if Re increased. On the other hand, if the Gr number increased the bouncy force will be dominated, and two strong vortices will appear in the cavity if Re is low. Generally, in this case, a two-symmetric-vortex flow pattern is formed. The results are obtained for wide ranges of Ra, Re and φ. It was concluded that:

• This phenomenon can be ascribed to the fact that the lid-driven becomes more active to enhance the heat transfer. Where Non-uniform heating and arc cavity are a good case to enhance the heat transfer.
• The Nusselt number increases with increased Ra or Re or φ. Where flow strength was increases as φ increasesed for all values of Ra or Re.
• The maximum heat transfer rate is obtained by the nanofluid which its effect is more pronounced at high Rayleigh numbers.
• The isotherms are regulated near the arc-shape wall causing a steep temperature gradient at these regions.
• Correlation equations of average Nusselt number from numerical results are presented.
• At a high Ra/Re ratio, the effects of natural convection are dominant, where $Nu_{ave}$ increases about 6 % at max. volume fraction.
• For future research work, the suggestion that multiphase modeling or multi-lid driven case.

## Declarations

### Author contribution statement

Sattar Aljabair: Conceived and designed the experiments; Analyzed and interpreted the data.

Ali L. Ekaid: Performed the experiments; Analyzed and interpreted the data.

Sahira Hasan Ibrahim: Contributed reagents, materials, analysis tools or data; Wrote the paper.

Israa Alesbe: Performed the experiments; Wrote the paper.

### Funding statement

This research did not receive any specific grant from funding agencies in the public, commercial, or not-for-profit sectors.

### Data availability statement

Data will be made available on request.

### Declaration of interests statement

The authors declare no conflict of interest.

### Additional information

No additional information is available for this paper.
